# Altered tubulin detyrosination due to SVBP malfunction induces cytokinesis failure and senescence, underlying a complex hereditary spastic paraplegia

**DOI:** 10.1111/acel.14355

**Published:** 2024-10-16

**Authors:** Nathalie Launay, Maria Espinosa‐Alcantud, Edgard Verdura, Gorka Fernández‐Eulate, Jon Ondaro, Pablo Iruzubieta, Maria Marsal, Agatha Schlüter, Montserrat Ruiz, Stephane Fourcade, Agustí Rodríguez‐Palmero, Miren Zulaica, Andone Sistiaga, Garazi Labayru, Pablo Loza‐Alvarez, Alejandro Vaquero, Adolfo Lopez de Munain, Aurora Pujol

**Affiliations:** ^1^ Neurometabolic Diseases Laboratory Institut d'Investigació Biomèdica de Bellvitge (IDIBELL), Hospital Duran i Reynals Barcelona Spain; ^2^ Center for Biomedical Research on Rare Diseases (CIBERER U759) Ministry of Science Innovation and University Madrid Spain; ^3^ Chromatin Biology Laboratory Josep Carreras Leukaemia Research Institute Badalona Spain; ^4^ Nord‐Est/Ile‐de‐France Neuromuscular Reference Center Institute of Myology, Pitié‐Salpêtrière Hospital Paris France; ^5^ Department of Neurology, Hospital Universitario Donostia, OSAKIDETZA‐Department of Neurosciences University of the Basque Cpuntry San Sebastian Spain; ^6^ Department of Neurosciences Instituto Biodonostia San Sebastián Spain; ^7^ Center of Biomedical Research in Neurodegenerative Diseases (CIBERNED) CIBER, Ministry of Science, Innovation and University Madrid Spain; ^8^ Department of Medicine, School of Medicine University of Deusto Bilbao Spain; ^9^ ICFO‐Institut de Ciencies Fotoniques The Barcelona Institute of Science and Technology Castelldefels Spain; ^10^ Pediatric Neurology Unit, Department of Pediatrics University Hospital Germans Trias i Pujol, Autonomous University of Barcelona Badalona Spain; ^11^ Department of Personality, Assessment and Psychological Treatment Faculty of Psychology University of the Basque Country (UPV/EHU) San Sebastian Spain; ^12^ Catalan Institution of Research and Advanced Studies (ICREA) Barcelona Spain

**Keywords:** centrosome, cytokinesis failure, HSP, microtubule detyrosination, senescence, SVBP

## Abstract

Senescence, marked by permanent cell cycle arrest may contribute to the decline in regenerative potential and neuronal function, thereby promoting neurodegenerative disorders. In this study, we employed whole exome sequencing to identify a previously unreported biallelic missense variant in SVBP (p.Leu49Pro) in six patients from three unrelated families. These affected individuals present with a complex hereditary spastic paraplegia (HSP), peripheral neuropathy, verbal apraxia, and intellectual disability, exhibiting a milder phenotype compared to patients with nonsense SVBP mutations described previously. Consistent with SVBP's primary role as a chaperone necessary for VASH‐mediated tubulin detyrosination, both patient fibroblasts with the p.Leu49Pro mutation, and HeLa cells harboring an SVBP knockdown exhibit microtubule dynamic instability and alterations in pericentriolar material (PCM) component trafficking and centrosome cohesion. In patient fibroblasts, structural abnormalities in the centrosome trigger mitotic errors and cellular senescence. Notably, premature senescence characterized by elevated levels of p16INK4, was also observed in patient peripheral blood mononuclear cells (PBMCs). Taken together, our findings underscore the critical role of SVBP in the development and maintenance of the central nervous system, providing novel insights associating cytokinesis failure with cortical motor neuron disease and intellectual disability.

## Graphical abstract

SVBP deficiency induces centrosome defects and premature senescence, promoting neurodegeneration. SVBP variant, SVBP‐KO, or pharmacological SVBP inhibition causes microtubule instability and PCM/centrosome disorganization, leading to reduced astral MTs, aberrant spindle formation, and cytokinesis failure. Cells expressing pathogenic SVBP may bypass the centrosome p53 checkpoint, leading to micro‐ and multinucleated cell accumulation. These nondiploid cells can cease to proliferate, and exhibit a senescence phenotype that may be deleterious for neuronal homeostasis.

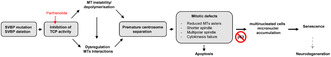

**Abbreviations**
AD: Alzheimer's disease
ALS: amyotrophic lateral sclerosis
CNS: the central nervous system
CSVS: The Collaborative Spanish Variant Server
CTL: control
ER: the endoplasmic reticulum
FBS: foetal bovine serum
FRDA: Friedreich ataxia
FTD: Frontotemporal dementia
HSP: Hereditary Spastic Paraplegias
LOD: combined logarithm of odds
MS: multiple sclerosis
MTs: microtubules
PACT‐RFP: the RFP‐tagged PACT domain
PBMCs: peripheral blood mononuclear cells
PD: Parkinson's disease
Peric: Pericentrin
PN: Parthenolide
siRNAs: small interfering RNAs
SNPs: single nucleotide polymorphisms
SVBP: small vasohibin‐binding protein
TLL: tubulin tyrosine ligase
Veh: vehicle
WES: whole exome sequencing
β‐gal: β‐galactosidase

## 1 INTRODUCTION

Hereditary spastic paraplegias (HSP) are a group of inherited neurodegenerative disorders characterized by the degeneration of the long descending axons of the corticospinal upper motor neurons, resulting in spasticity and weakness in the lower limbs (Fink, 2014). The clinical and genetic heterogeneity of HSP reflects the involvement of diverse cellular pathways, encompassing membrane and cargo trafficking, mitochondrial function, organelle shaping, lipid metabolism, and autophagy (Blackstone, 2018; Lo Giudice et al., 2014). Among these cellular pathways, microtubules (MTs) emerge as a crucial element due to several factors: (i) their regulation directly influences intracellular transport, (ii) they interact with organelles such as the endoplasmic reticulum (ER) and mitochondria, and (iii) mutations affecting proteins that directly interact with MTs (such as SPAST, KIF5A, KIF1A, REEP1, and REEP2) are present in approximately half of patients with genetically confirmed HSP (Blackstone, 2018; Lo Giudice et al., 2014).

Microtubules are dynamic polymers composed of α/β tubulin dimers that serve as “railways” for motor‐driven intracellular transport. They play a vital role in intracellular organization and chromosome segregation. MTs exhibit high dynamics, with their growth and shrinkage regulated by (1) the addition and loss of α‐ and β‐tubulin subunits, and (2) various types of posttranslational modifications (Janke, 2014). These modifications, such as detyrosination/tyrosination, acetylation, glycosylation, and (poly) glutamylation, collectively form a “tubulin‐code” that regulates interactions with molecular motors and other MT‐binding proteins (Janke, 2014; McKenna et al., 2023).

Patients with loss‐of‐function mutations in SVBP (small vasohibin‐binding protein) were reported to exhibit symptoms such as ataxia, intellectual disability, microcephaly, and muscular hypotonia (Iqbal et al., 2019; Pagnamenta et al., 2019). SVBP interacts with VASH proteins in a chaperone‐like manner, serving as a critical factor for the solubility/secretion and detyrosination activity of VASH1 and VASH2 (Aillaud et al., 2017; Nieuwenhuis et al., 2017). Detyrosinated tubulin is abundantly present in neuronal MTs and other long‐lived MT populations, where it plays a crucial role in axonal transport by facilitating the trafficking of kinesin‐1 (Konishi & Setou, 2009). Defective detyrosination caused proliferative defects during neurogenesis, leading to microcephaly and abnormal behavior (Landskron et al., 2022). Moreover, spindle detyrosination is crucial for the accurate chromosome congression and symmetry breakage during female meiosis, underscoring the essential role of MT detyrosination in maintaining genomic stability (Barisic et al., 2015).

In this study, we have identified a novel biallelic missense variant in SVBP among six individuals exhibiting spastic paraparesis accompanied by sensorimotor axonal neuropathy, verbal apraxia, epilepsy, and intellectual disability, thereby expanding the previously reported phenotype. Our findings, based on analyses of patient‐derived fibroblasts and SVBP knockdown cells, reveal a pivotal role of SVBP in centrosome cohesion and chromosome segregation. Moreover, our results shed light for the first time on the potential involvement of cellular senescence as a driver of these neurological disorders.

## 2 RESULTS

### 2.1 Variant identification and clinical features

Through whole exome sequencing (WES), we have identified a consistent biallelic SVBP variant in six individuals belonging to three unrelated families originating from the same geographic region in Northern Spain. The novel missense variant in the SVBP gene (Chr1:43273140A > G, NM_199342: exon3: c.146 T > C, p.Leu49Pro) is not present in control databases such as ExAC and 1000 genomes, with only two heterozygous carrier individuals recorded in the GnomAD database. Furthermore, this variant is absent from the Collaborative Spanish Variant Server (CSVS), which includes sequence data from 1644 unrelated individuals. All single nucleotide polymorphisms (SNPs) within the homozygous genomic region shared by the patients sequenced by WES (spanning at least 2.32 Mb) exhibit identical genotypes in the five analyzed patients (families A and B). This observation suggests that the variant is located within the same haplotype, indicating it as a founder variant. Segregation analysis in all unaffected members of both families supports the recessive mode of inheritance previously reported for SVBP. The combined logarithm of odds (LOD) score, considering cosegregation data from all genotyped individuals, reached 2.73.

**FIGURE 1:**
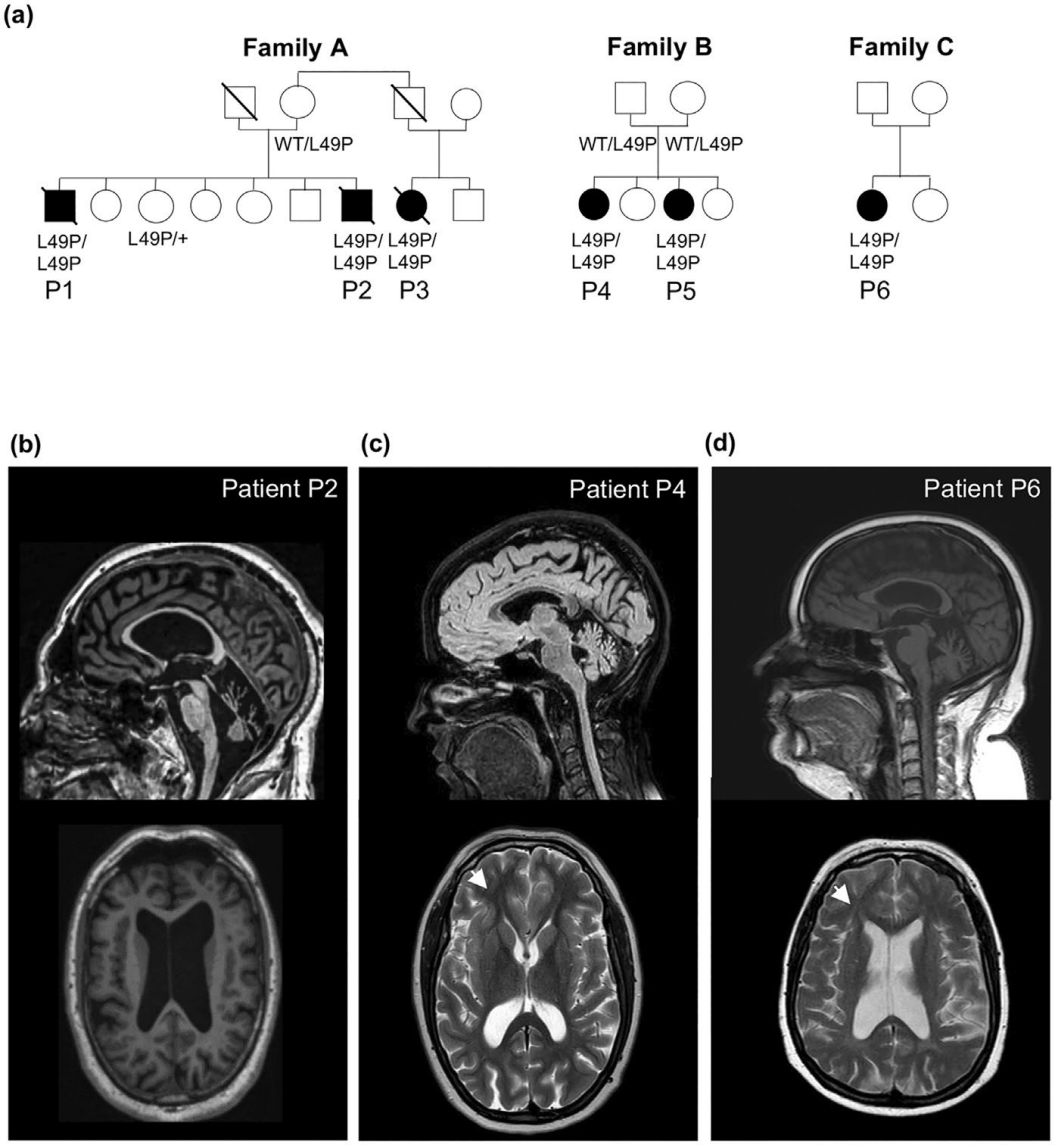
Clinical features of families with a novel *SVBP* mutation. (a) Pedigree for families A, B, and C. (b) Axial and sagittal T1 MRI sequences of patient P2 showing diffuse cerebellar atrophy, enlarged ventricles, thin corpus callosum, and diffuse cerebellar atrophy (c) Sagittal T1 and axial FLAIR MRI of patient P4 showing cerebellar vermis atrophy, ears of the lynx sign (white arrow), an asymmetrical ventricle enlargement. (d) Axial T2 and sagittal T1 MRI sequences from patient P6 showing ears of the lynx sign (white arrow), enlarged ventricles, and vermis cerebellar atrophy.

Patients from families A, B, and C presented at birth or childhood with neurodevelopmental delays and later exhibited intellectual disabilities. Subsequently, they developed a complex spastic paraplegia syndrome associated with verbal apraxia and axonal neuropathy (Figure 1a). The clinical characteristics of all six patients are summarized in Table 1. Brain MRIs of patients P2, P4, and P6 were analyzed, revealing several shared features. All three exhibited corpus callosum thinning, cerebellar atrophy, and ventriculomegaly (Figure 1b–d). Additionally, two patients displayed frontal periventricular hyperintensities on T2 imaging, resembling the “ear of the lynx” sign (Figure 1c,d). Notably, half of the patients have a history of cancer, all of which are of epithelial origin.

**TABLE 1:** Main clinical features of families A, B, and C.

Family	A			B		C
Patient	P1	P2	P3	P4	P5	P6
Mutation	c.146 T > C Hom p. Leu49Pro	c.146 T > C Hom p. Leu49Pro	c.146 T > C Hom p. Leu49Pro	c.146 T > C Hom p. Leu49Pro	c.146 T > C Hom p. Leu49Pro	c.146 T > C Hom p. Leu49Pro
Gender	M	M	F	F	F	F
Ethnicity	Caucasian	Caucasian	Caucasian	Caucasian	Caucasian	Caucasian
Parental Consanguinity	No	No	No	No	No	No
First symptoms and age of onset	Neurodevelopmental delay since birth	Neurodevelopmental delay since birth	Neurodevelopmental delay since birth	Mild clumsy gait and foot deformity since childhood	Neurodevelopmental delay since birth	Neurodevelopmental delay since birth
Psychomotor development	Delayed	Delayed	Delayed	N/A	Delayed	Delayed
Intellectual disability	+	+	+	+	+	+
Motor examination	Spastic paraparesis Areflexia Wheelchair‐bound (adult)	Spastic paraparesis Bradykinesia, distal lower limb weakness Wheelchair‐bound (adult)	Spastic paraparesis	Spastic paraparesis Distal lower limb amyotrophy and weakness, ankle areflexia	Spastic paraparesis Distal lower limb weakness, areflexia	Ataxic‐spastic gait. Brisk reflexes except for Achilles reflex (absent)
Ataxia	+	+	N/A	+	−	+
Verbal apraxia	−	+	−	+	+	N/A
Epilepsy	−	+	+	N/A	+	−
Aggressive behavior	−	+	+	−	−	+
Ophthalmologic manifestations	−	Strabismus, Nystagmus	N/A	−	Nystagmus	Nystagmus
Other clinical manifestations	Hammer toes Adenocarcinoma with hepatic metastasis. Deceased at age 59 years	Scoliosis, lumbar hyperlordosis, *pes planus*, Hammer toes Colonic tubular adenoma Deceased at 52 years	Deceased at age 59 years	*Pes cavus*, Hammer toes Feet hypoalgesia and hypopallesthesia Cold, erythematous feet		*Pes cavus*. Hammer toes. Hypopallesthesia. Breast cancer (at 51 years)
MRI	N/A	Cerebellar atrophy, ventriculomegaly, middle cerebellar peduncles atrophy. Cavum septum pellucidum persistence	N/A	Brain atrophy, ventriculomegaly. Ear of the lynx sign	N/A	Cerebellar atrophy, ventriculomegaly, Ear of the lynx sign
Nerve Conduction Studies (NCS)	N/A	Lower limb axonal sensorimotor neuropathy	N/A	Axonal sensorimotor neuropathy	Axonal sensorimotor neuropathy	Axonal sensorimotor neuropathy

Abbreviations: F, female; M, male; N/A, not available; Y, years.

### 2.2 *SVBP* variant alters SVBP expression and impairs MT detyrosination

**FIGURE 2:**
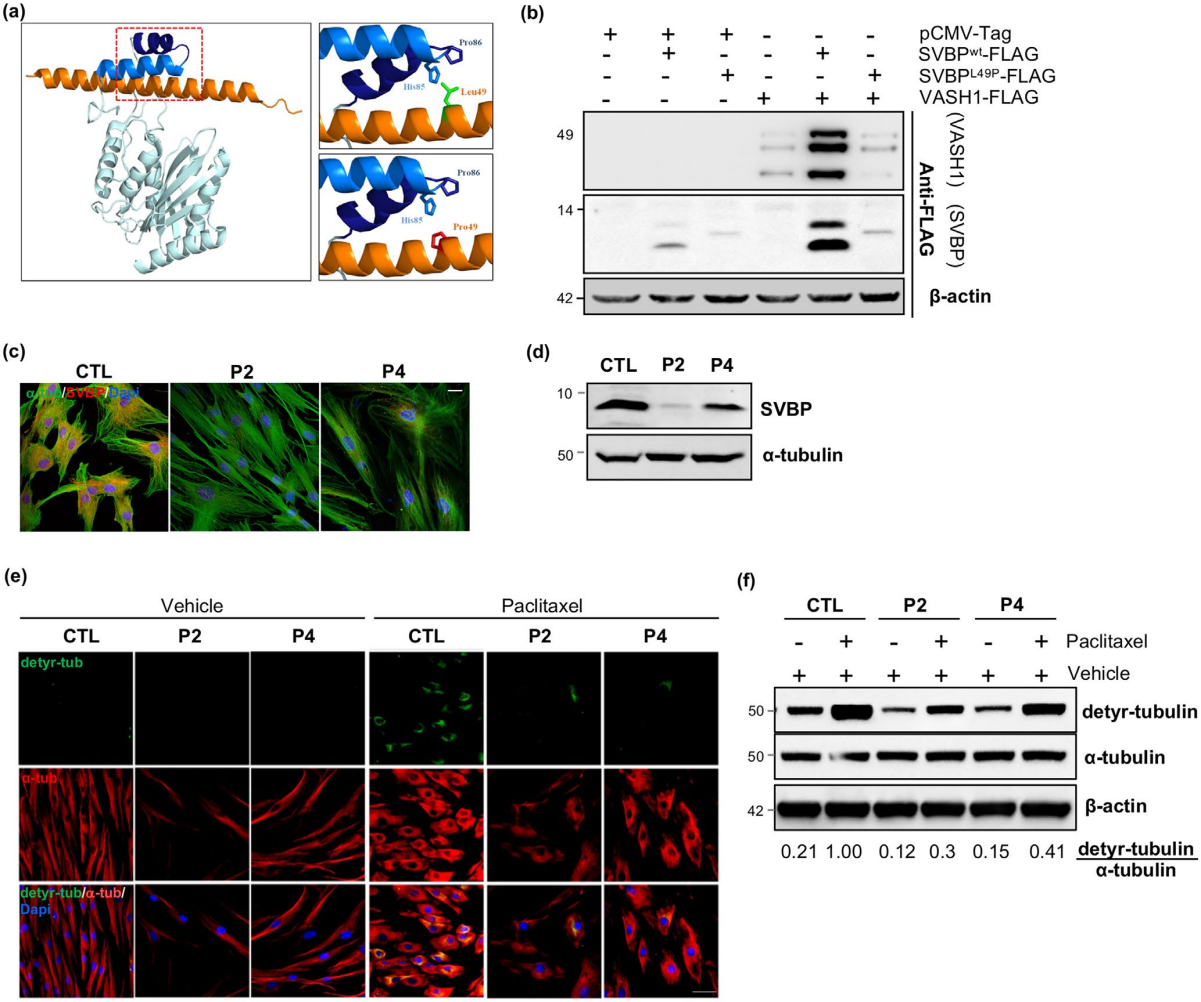
Pathogenicity of *SVBP* variant on the VASH1 secretion and MT detyrosination activity. (a) Close‐up views of the VASH1–SVBP interface, with interacting residues shown as sticks. VASH1 residues are colored blue and labeled with blue letters, while SVBP residues are colored orange and labeled with orange letters. (b) HeLa cells transfected with vectors directing the expression of FLAG‐tagged SVBP^WT^, SVBP^L49P^ and VASH1, or combinations thereof, were subjected to immunoblot analysis with anti‐Flag antibody. Total amounts of β‐actin were used as a loading control. (c) Control (CTL) and patient (P2 and P4) fibroblasts were stained with anti‐SVBP (red) and anti‐α‐tubulin (α‐tub; green) antibodies and DAPI (blue). Scale bars: 10 μm. (d) Control (CTL) and patient (P2 and P4) fibroblasts were subjected to immunoblot analysis using the anti‐SVBP antibody. Total amounts of α‐tubulin were used as a loading control. (e) Control (CTL) and patient (P2 and P4) fibroblasts were treated with vehicle or paclitaxel and stained with anti‐α‐tubulin (α‐tub; red) and anti‐detyrosinated tubulin (detyr‐tub; green) antibodies and DAPI (blue). Scale bars: 50 μm. (f) Control (CTL) and patient (P2 and P4) fibroblasts were treated with vehicle or paclitaxel and subjected to immunoblot analysis using antibodies directed against detyrosinated (detyr‐tubulin) and α‐tubulin. Total amounts of β‐actin were used as a loading control. The relative ratios of detyrosinated versus total ⍺‐tubulin levels are indicated (*n* = 3).

Our in silico structural analysis revealed that the p.Leu49Pro substitution may disrupt intramolecular hydrophobic interactions and/or destabilize the conserved α‐helical conformation of SVBP. This conformational change is crucial for its interaction with VASH1 and the detyrosination of the α‐tubulin peptide (Liao et al., 2019) (Figure 2a). To validate this prediction, we cotransfected HeLa cells with equimolar amounts of C‐terminal Flag‐tagged human VASH1 and either wild‐type or p.Leu49Pro mutant C‐terminal Flag‐tagged human SVBP. Subsequently, we analyzed the expression levels of VASH1 and SVBP by Western blot. In contrast to wild‐type SVBP, the mutant SVBP protein was undetectable in the cell lysate (Figure 2b), suggesting possible degradation of the SVBP variant. Moreover, a significant reduction in three posttranslational forms of VASH1 (42 kDa, 36 kDa, and 32 kDa) was observed in cells transfected with the mutant SVBP (Figure 2b), strongly indicating that the SVBP mutation impairs VASH1 secretion and/or solubility. Immunofluorescence and Western blot analyses demonstrated decreased SVBP levels in patient fibroblasts, further confirming that the p.Leu49Pro mutation affects protein stability and leads to its degradation (Figure 2c,d).

To investigate the potential impact of the SVBP mutant on MT detyrosination, we employed paclitaxel to elevate detyrosinated tubulin levels in fibroblasts. Paclitaxel functions by stabilizing MTs and removing the free α/β‐tubulin dimers, which serve as substrates for tubulin tyrosine ligase (TTL) (Prota et al., 2013). Immunofluorescence and Western blot experiments revealed a significant reduction in detyrosinated α‐tubulin levels in patient fibroblasts compared to controls (Figure 2e,f), providing evidence that the SVBP mutation disrupts MT detyrosination. Collectively, these findings underscore the critical role of residue Leu49 in SVBP for the interaction with VASH1, suggesting that its substitution with Pro hampers MT detyrosination by reducing the abundance of active SVBP‐VASH1 heterodimers.

### 2.3 *SVBP* mutant induces MT dynamic instability and centrosome cohesion deficit

**FIGURE 3:**
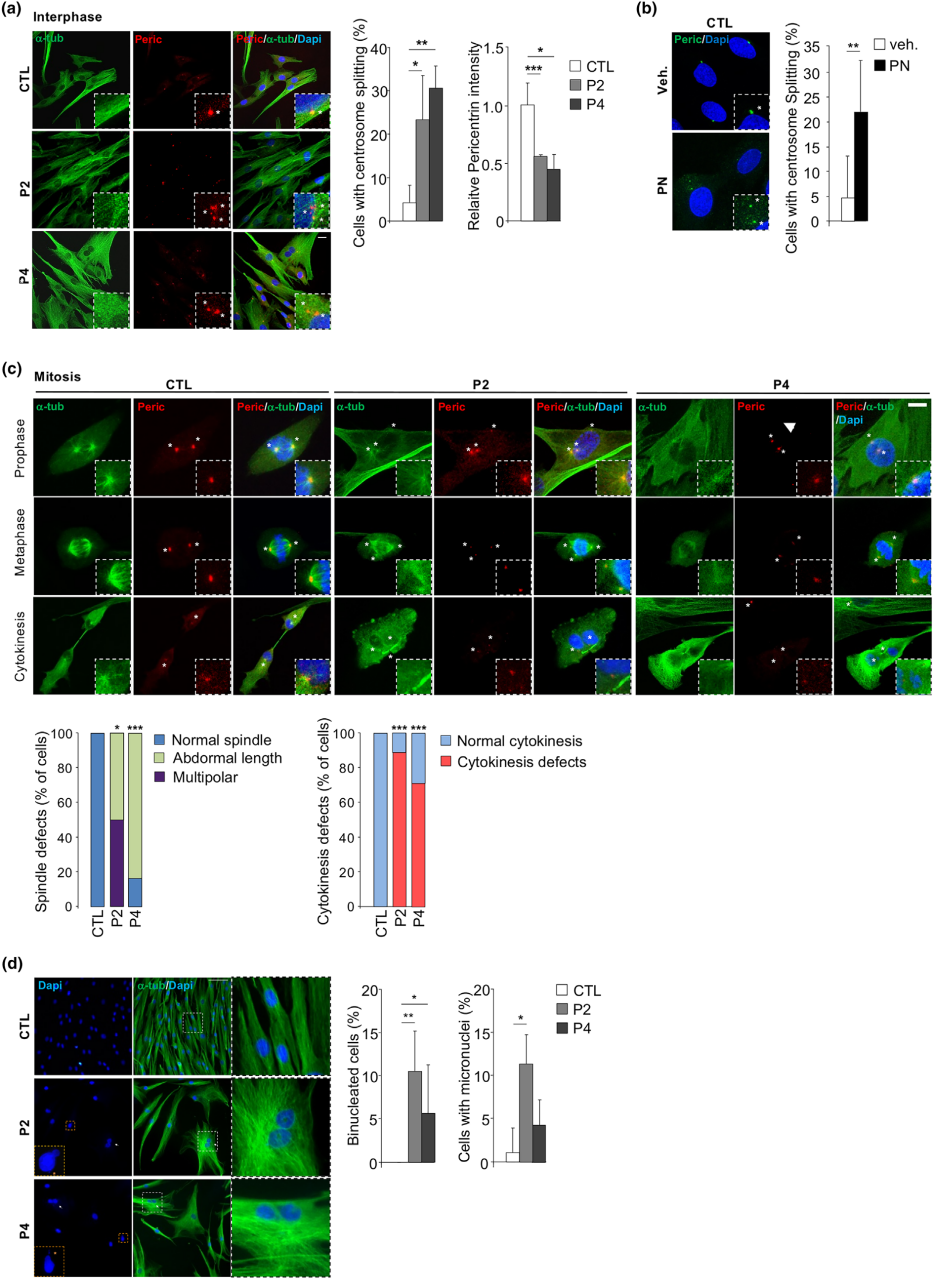
*SVBP* mutant induces centrosome cohesion deficit, mitotic spindle abnormalities, cytokinesis failure, and chromosome instability. (a) Interphase control (CTL) and patient (P2 and P4) fibroblasts were stained with alpha‐tubulin (α‐tub; green) and anti‐pericentrin (Peric.; red) antibodies and DAPI (blue) at cell passages <10 (inset shows enlargement of the PCM, asterisk (*) mark the position of centrosome). Scale bars, 10 μm. Percentage of control (CTL) and patient fibroblasts (P2 and P4) with split centrosomes and centrosomal pericentrin intensity were quantified. *n* ≥ 50 cells/100 centrosomes; mean ± SD; *p < 0.05; **p < 0.01 by one‐way ANOVA with Tukey post hoc tests. (b) Representative picture of control fibroblasts (CTL) treated with vehicle (Veh.) or parthenolide (PN, 5 μM, 24 h) and stained with anti‐pericentrin (Peric.; green) antibodies and DAPI (blue) (white arrows indicate the position of the centrosome). Scale bars, 20 μm. Percentage of cells treated or not with parthenolide (PN) was quantified. *n* ≥ 20 cells/40 centrosomes; mean ± SD; **p < 0.01 by two‐tailed *t* test. (c) Control (CTL) and patient (P2 and P4) fibroblasts were stained at different mitotic phases (prophase, metaphase, and cytokinesis) with alpha‐tubulin (α‐tub; green) and anti‐pericentrin (Peric.; red) antibodies and DAPI (blue) at cell passages <10 (inset shows enlargement of the PCM, asterisk (*) mark the position of centrosome). Scale bars, 5 μm. Mitotic defects (spindle abnormalities and cytokinesis defects) were quantified in control (CTL) and patient (P2 and P4 fibroblasts). *n* ≥ 10 cells for each mitotic phase; Mean ± SD; *p < 0.05; *** < *p* < 0.001 by one‐way ANOVA with Tukey post hoc tests. (d) Representative pictures of control (CTL) and patient (P2 and P4) fibroblasts at cell passages >10 stained with anti‐α‐tubulin (α‐tub; green) and DAPI (blue), showing the presence of micronuclei and binucleated cells (white arrows) (insert shows enlargement of the indicated area). Scale bars, 50 or 10 μm. The percentage of cells with micronuclei and binucleated cells were quantified. *n* ≥ 50; Mean ± SD; *p < 0.05; **p < 0.01 by one‐way ANOVA with Tukey post hoc tests.

Given the established association between high levels of MT detyrosination and centrioles (Janke, 2014; Song & Brady, 2015), suggesting a pivotal role of MT detyrosination in centrosome stabilization and integrity, we opted to investigate the impact of the SVBP mutation on centrosome cohesion. Intriguingly, immunofluorescence analysis revealed premature centrosome separation in patient fibroblasts during interphase. The proportion of cells exhibiting centrosomes separated by >2 μm increased from 4% in controls to >20% in patient cells (Figure 3a). Consistent with these findings, parthenolide, an inhibitor of MT detyrosination (Freund et al., 2020), induced abnormal centriole separation in nearly 20% of control fibroblasts (Figure 3b), mirroring the observed ratio in patient cells.

Subsequently, we quantified pericentrin levels to evaluate centrosome integrity. Our analysis revealed a significant reduction in centrosomal pericentrin staining in patient fibroblasts compared to controls (Figure 3a), indicative of disrupted pericentriolar material (PCM) assembly due to the SVBP mutation. Consistently, high‐resolution fluorescence microscopy unveiled an increase in peripheral and cytoplasmic pericentrin foci along the MT cytoskeleton in interphase patient fibroblasts, suggesting a defect in MT‐dependent trafficking where PCM components are inadequately transported to centrosomes (Figure S1). Furthermore, we observed MT depolymerization and degradation in patient fibroblasts, evident from the punctuated pattern of MT staining (Figure S1). Consequently, we conclude that the SVBP variant induces MT dynamic instability, leading to alterations in PCM component trafficking and a deficit in centrosome cohesion.

### 2.4 *SVBP* mutant leads to spindle morphology alteration, cytokinesis failure, and chromosome instability

The PCM serves as a crucial hub for MT nucleation and regulates the number and composition of MTs throughout the cell cycle (Zimmerman et al., 2004). Consistently, studies employing small interfering RNAs (siRNAs) targeting both pericentrin isoforms (A and B) have demonstrated a reduction in astral MTs and mitotic spindle length in SAOS cells (Zimmerman et al., 2004). Correspondingly, our analysis revealed that patient fibroblasts exhibited shorter mitotic spindles and impaired formation of MT asters (Figure 3c), suggesting that the SVBP mutation alters MT nucleation. These observations align with a recent study demonstrating that depletion of vasohibin in U2OS cells resulted in shorter mitotic spindles, accompanied by a significant reduction in astral MTs (Liao et al., 2019).

Remarkably, our immunofluorescence experiments uncovered that 80% of patient fibroblasts experienced cytokinesis failure, evidenced by intercellular cytoplasmic bridges and the presence of binucleated cells (Figure 3c,d). Furthermore, the elevated rate of micronucleus formation (~10%) observed in patient fibroblasts compared to control cells indicated compromised chromosome stability (Figure 3d). Collectively, these findings suggest a crucial role of SVBP in ensuring accurate mitosis and establish a link between SVBP deficiency and alterations in spindle morphology, cytokinesis failure, and chromosome instability.

### 2.5 CRISPR/Cas9‐mediated knockout of *SVBP* in HeLa cells replicates centrosome cohesion deficit and mitosis abnormalities

**FIGURE 4:**
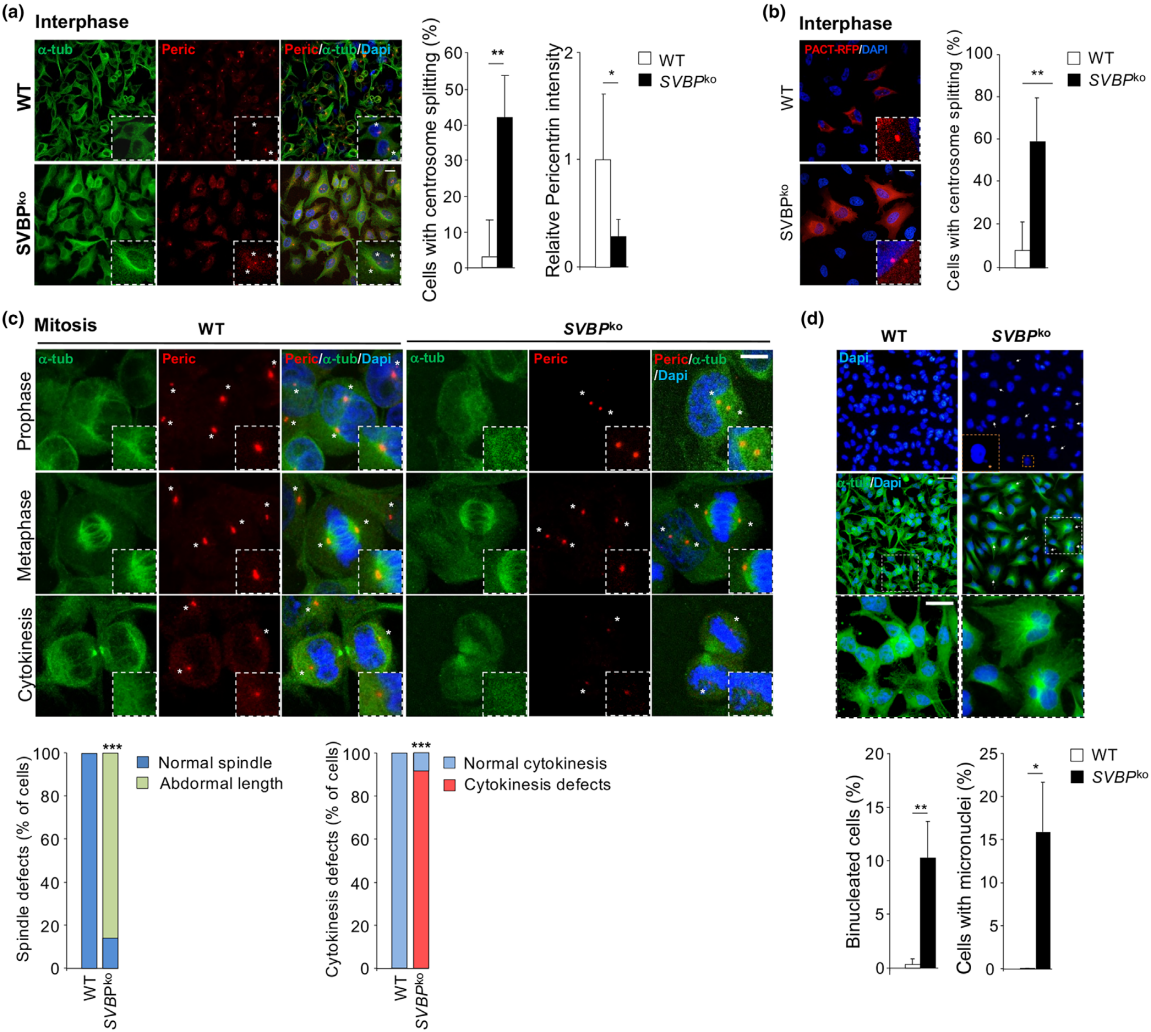
CRISPR/Cas9 knockout of *SVBP* in HeLa cells induces centrosome abnormalities and aberrant mitosis. (a) Interphase Wild‐type (WT) and *SVBP*‐KO (*SVBP* ^ko^) HeLa cells were stained with alpha‐tubulin (α‐tub; green) and anti‐pericentrin (Peric.; red) antibodies and DAPI (blue) (inset shows enlargement of the PCM; the asterisk (*) marks the position of the centrosome). Scale bars, 10 μm. Percentage of wild‐type (WT) and *SVBP*‐KO (*SVBP* ^ko^) HeLa cells with split centrosomes and centrosomal pericentrin intensity were quantified. *n* ≥ 30 cells/60 centrosomes per condition; Mean ± SD; **p < 0.01 by two‐tailed *t* test. (b) Representative image of wild‐type (WT) and *SVBP*‐KO (*SVBP* ^ko^) expressing PACT‐RFP (asterisk (*) mark the position of centrosome). Twenty‐four hours after transfection, cells were fixed and stained for DNA content (DAPI; blue). Scale bars, 10 μm. Percentage of wild‐type (WT) and *SVBP*‐KO (*SVBP* ^ko^) HeLa cells expressing PACT‐RFP with split centrosomes was quantified. *n* ≥ 10 cells/20 centrosomes per condition; Mean ± SD; **p < 0.01 by two‐tailed *t* test. (c) Wild‐type (WT) and *SVBP*‐KO (*SVBP* ^ko^) HeLa cells were stained at different mitotic phases (prophase, metaphase, and cytokinesis) with alpha‐tubulin (α‐tub; green) and anti‐pericentrin (Peric., red) antibodies and DAPI (blue) (inset shows enlargement of the PCM; the asterisk (*) marks the position of the centrosome). Mitotic defects (spindle abnormalities and cytokinesis defects) in wild‐type (WT) and *SVBP*‐KO (*SVBP* ^ko^) HeLa cells were quantified. *n* ≥ 10 cells for each mitotic phase; Mean ± SD; *p < 0.05; *** < *p* < 0.001 by two‐tailed *t* test. (d) Representative pictures of wild‐type (WT) and *SVBP*‐KO (*SVBP* ^ko^) HeLa cells stained with anti‐alpha‐tubulin (α‐tub, green) and DAPI (blue) at passages >10, showing the presence of multinucleated cells (white arrows indicate multinucleated cells); insert shows enlargement of the indicated area. Scale bars, 50 or 10 μm. The percentage of multinucleated and micronucleated wild‐type (WT) and *SVBP*‐KO (*SVBP* ^ko^) HeLa cells were quantified. *n* ≥ 50; Mean ± SD; *p < 0.05; **p < 0.01 by two‐tailed *t* test.

To confirm that the observed defects in patient fibroblasts were indeed attributed to SVBP deficiency, we employed the CRISPR/Cas9 technique to generate an SVBP knockout in HeLa cells (Figure S2). Consistent with our earlier findings, we noted a significant increase in centrosome splitting and a reduction in centrosomal pericentrin staining during interphase in SVBP‐KO HeLa cells compared to wild‐type cells (Figure 4a). Similar outcomes were observed when SVBP‐KO HeLa cells were transfected with an expression vector encoding the RFP‐tagged PACT domain (PACT‐RFP) (Figure 4b), a conserved motif known to target centrosomes by tagging AKAP450 and pericentrin (Gillingham & Munro, 2000). Additionally, SVBP‐KO HeLa cells displayed a shorter mitotic spindle size, reduced astral MTs, cytokinesis failure, and increased micronuclei formation (Figure 4c,d), collectively mimicking the phenotype observed in patient fibroblasts.

### 2.6 *SVBP* mutant induces cell cycle arrest and senescence

**FIGURE 5:**
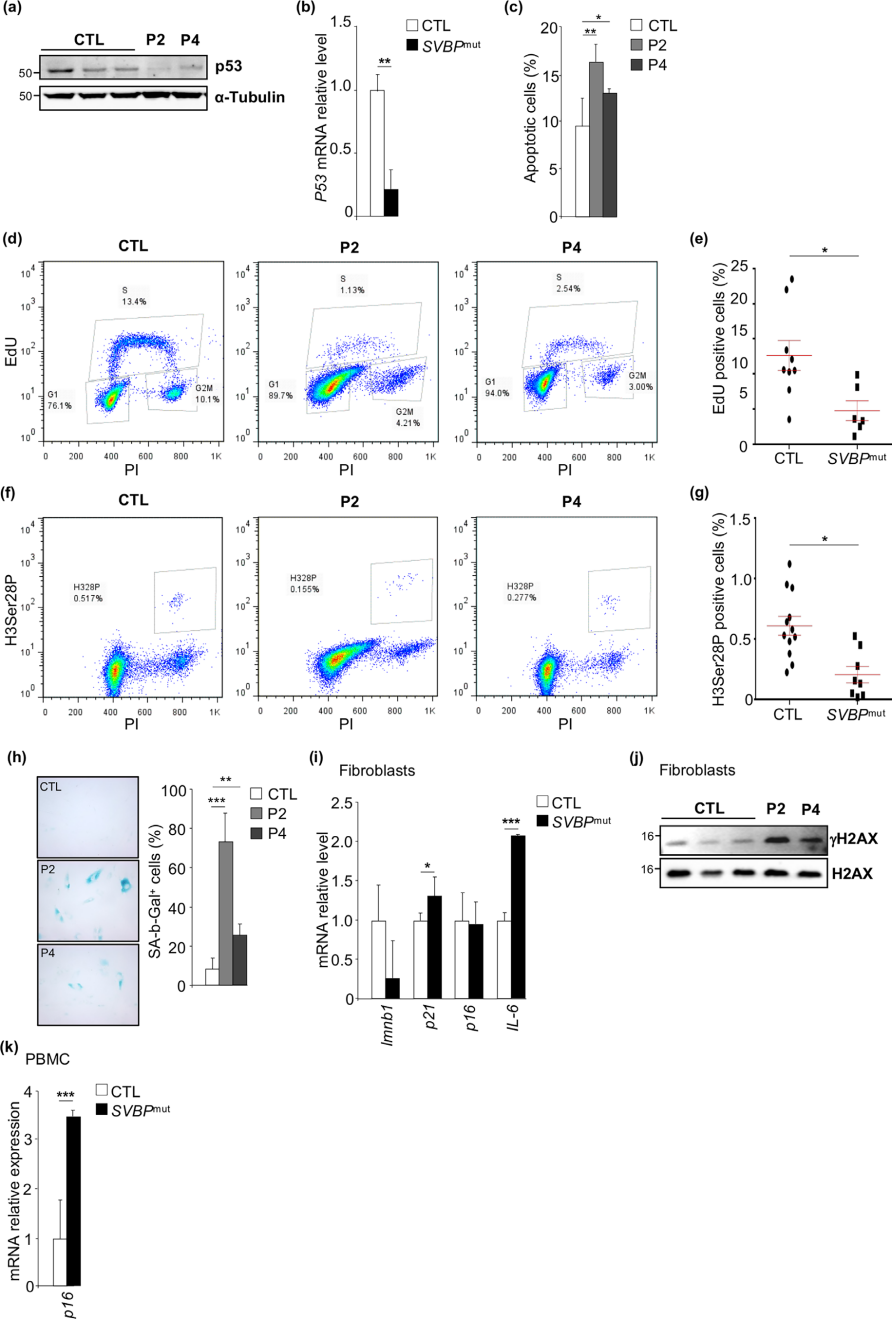
*SVBP* mutant induces cell cycle arrest and senescence. (a) Control (CTL) and patient (P2 and P4) fibroblasts at cell passage >10 were subjected to immunoblot analysis using anti‐p53 antibodies. Total amounts of α‐tubulin were used as a loading control. (b) Quantitative RT‐PCR analysis of *p53* gene expression in control (CTL) and patient (*SVBP* ^mut^) fibroblasts at cell passage >10. Two independent experiments were performed. *n* = 3–2, Mean ± SD; **p < 0.01 by two‐tailed *t* test. (c) Representative histogram from flow cytometry of control (CTL) and patient fibroblasts (*SVBP* ^mut^) positive for Annexin V‐APC at cell passage >10. Two independent experiments were performed. *n* = 3–1, Mean ± SD; *p < 0.05; **p < 0.01 by one‐way ANOVA with Tukey post hoc tests. (d) Representative flow cytometry profiles of control (CTL) and patient (*SVBP* ^mut^) fibroblasts labeled with EdU at cell passage >10. (e) Quantification of EdU positive cells. Two independent experiments were performed. *n* = 3–2, Mean ± SD; *, *p* < 0.05 by two‐tailed *t* test. (f) Representative flow cytometry profiles of control (CTL) and patient (*SVBP* ^mut^) fibroblasts labeled with H3Ser28P at cell passage >10. (g) Quantification of H3Ser28P positive cells. Two independent experiments were performed. *n* = 3–2, Mean ± SD; *, *p* < 0.05 by two‐tailed *t* test. (h) Representative pictures of control (CTL) and patient (P2 and P4) fibroblasts at cell passage >10 stained for SA‐β‐gal activity. The percentage of SA‐β‐gal‐positive cells was quantified. Two independent experiments were performed. *n* = 3–1, Mean ± SD; **p < 0.01; *** < *p* < 0.001 by one‐way ANOVA with Tukey post hoc tests. (i) Quantitative RT‐PCR analysis of *Lmnb1*, *p21*, *p16*, and *IL‐6* gene expression in control (CTL) and patient (*SVBP* ^mut^) fibroblasts at cell passage >10. Two independent experiments were performed. *n* = 3–2, Mean ± SD; *, *p* < 0.05; *** < *p* < 0.001 by two‐tailed *t* test. (j) Control (CTL) and patient (P2 and P4) fibroblasts at cell passage >10 were subjected to immunoblot analysis using anti‐γH2AX and anti‐H2AX antibodies. (k) Quantitative RT‐PCR analysis of, *p16* gene expression in control (CTL) and patient (*SVBP* ^mut^) PBMC. Two independent experiments were performed. *n* = 17–20, Mean ± SD; *p < 0.05; **p < 0.01; *** < *p* < 0.001 by two‐tailed *t* test.

Centrosome alterations or mitotic errors typically activate the apoptosis machinery through p53 (Imreh et al., 2016). Therefore, we examined p53 expression in patient's fibroblasts by Western Blot analysis. While no change was observed in early cell passages (<10), the decreased protein and mRNA expression levels of p53 in patient fibroblasts at cell passage >10 suggested the progressive inactivation of the p53‐dependent apoptosis response (Figure 5a,b; Figure S3a,b). Consistently, flow cytometry apoptosis assays showed a slight increase in apoptotic cell levels in patient fibroblasts at late passage compared to controls (Figure 5c), indicating that apoptosis is a minor outcome in SVBP mutant cells.

We then investigated the impact of the SVBP mutation on cell proliferation. EdU incorporation and histone H3‐Serine 28 phosphorylation levels (H3Ser28P) were used to monitor S phase progression and the late‐G(2)/M status of cells, respectively. Flow cytometry analysis revealed equivalent EdU incorporation in early passages of control and patient fibroblasts (Figure S3c). However, by cell passages >10, patient fibroblasts displayed decreased EdU incorporation compared to control fibroblasts (Figure 5d,e). Similarly, we found that the phosphorylation of H3Ser28 decreased significantly from cell passages >10 in patient fibroblasts (Figure 5f,g; Figure S3d). Therefore, our results indicated that patient fibroblasts progressively arrested in G1/G0 phase.

To assess senescence levels, we first performed β‐galactosidase (β‐gal) staining, which revealed increased senescence‐associated β‐galactosidase activity in patient cells, correlated with an increasing number of cell passages (Figure 5h). mRNA levels of *lmnb1* were decreased while *p21* levels were raised, consistent with augmented β‐galactosidase staining with increasing cell passages (Figure 5i). Remarkably, we observed a very high expression level of *IL‐6*, a senescence‐associated inflammatory mediator molecule (SASP) in low‐passage patient fibroblasts (Figure 5i, Figure S3c). Moreover, we observed a correlation between increased H2A histone (γ‐H2AX) phosphorylation, a marker of DNA damage (Biran et al., 2017), and the accumulation of senescent cells in patients (Figure 5j; Figure S3a).

To corroborate our in vitro findings, we assessed the expression of *CDKN2A/p16INK4* in peripheral blood mononuclear cells (PBMCs) from both control individuals and patients. *p16INK4* serves as a well‐established biomarker of senescence in various tissues, including T cells, where it restricts their replicative capacity (Y. Liu et al., 2009). Remarkably, the relative expression of *CDKN2A/p16INK4* was significantly higher in patient PBMCs (3.4 ± 0.1 fold) compared to controls (Figure 5k), aligning with the senescence phenotype observed in patient fibroblasts.

## 3 DISCUSSION

Using whole‐exome sequencing (WES), we uncovered a novel bi‐allelic SVBP variant in six individuals from three unrelated families. Our functional investigations provide compelling evidence suggesting that centrosome abnormalities and subsequent senescence may serve as underlying drivers of HSP. When considering the findings of Iqbal and Pagnamenta (Iqbal et al., 2019; Pagnamenta et al., 2019), along with our cohort of six patients, a collection of common clinical features emerges. All patients exhibited intellectual disability along with delayed gross motor and speech development. Notably, patients harboring the missense p.Leu49Pro variant manifested a motor‐predominant phenotype characterized by complex HSP and axonal neuropathy, significantly impacting their mobility compared to previously reported individuals who presented with hypotonia, dysmorphia, and severe cognitive impairment. The earlier onset and more severe neurodevelopmental features observed in the previously reported cases could be attributed to their loss‐of‐function variants. From a neuroimaging perspective, several common features were observed among our patients, including thin corpus callosum, ears of the lynx sign, and enlarged ventricles or cerebellar atrophy.

These characteristics are also evident in the MRI findings of previously described SVBP patients (Iqbal et al., 2019; Pagnamenta et al., 2019), indicating a distinct pattern that could aid in clinical diagnosis and differentiate from other forms of HSP with corpus callosum thinning and ears of the lynx sign (Ebrahimi‐Fakhari et al., 2021; Pascual et al., 2019).

The implication of dysfunctional SVBP in inducing structural centrosome defects and premature centrosome separation strongly suggests a crucial role of MT detyrosination in the centrosome cycle. Dysfunctional centrosomes not only impair the long‐term proliferative capacity but also the polarization of neural stem cells, leading to conditions such as microcephaly and neurodegenerative disorders like Parkinson's disease (Goundiam & Basto, 2021; Madero‐Pérez et al., 2018). This is further supported by the fact that many genes associated with microcephaly encode centrosome proteins (CPAP, CEP152, CEP135, STIL, and CDK5RAP2) involved in centriole biogenesis and centrosome maturation (Naveed et al., 2018). Previous research by Madero‐Perez et al. has revealed that pathogenic LRRK2 in Parkinson's disease causes centrosomal polarity and cohesion deficits in both dividing and nondividing cells, resulting in impaired neurite outgrowth, cell polarization, and migration (Madero‐Pérez et al., 2018). Intriguingly, downregulation of SVBP has been shown to disrupt neuronal migration in the developing mouse neocortex (Pagnamenta et al., 2019). Furthermore, cultured neurons lacking SVBP displayed a clear delay in axon differentiation and severe morphological defects, suggesting that the brain atrophy observed in SVBP knockout mice likely originates from abnormal differentiation and maturation of deficient neurons (Aillaud et al., 2017). Hence, we propose that dysfunctional centrosomes and subsequent inaccurate cytokinesis in mitosis, induced by SVBP malfunction, may disrupt progenitor proliferation and polarity of neural cells, thereby interfering with the development and maintenance of the central nervous system (CNS).

Increasing evidence suggests a link between centrosome defects and aging, indicating that abnormalities in centrosomes may directly or indirectly trigger cellular senescence (Wu et al., 2020). Consequently, due to the increased frequency of abnormal mitosis and dysfunctional centrosomes, patient fibroblasts exhibit prominent features of senescence, including DNA damage, growth arrest, increased β‐galactosidase staining, decreased expression of *Lamin B1*, and elevated levels of *p21*. Notably, patient PBMCs display higher levels of *p16INK4a*, indicating premature aging of immune cells. Additionally, our findings suggest that inactivation of the p53 pathway may render cells less sensitive to DNA damage‐induced apoptosis, which is offset by increased senescence. Previous studies on mutations causing human microcephaly have implicated p53 activation in excessive apoptosis of neuronal cells and reduced brain size (Chen et al., 2014; Insolera et al., 2014; Phan et al., 2021). In some cases, inhibition of p53 in these mutants was able to increase cortical thickness, but in others, it worsened brain phenotypes (Insolera et al., 2014; Little & Dwyer, 2019), suggesting that a broad spectrum of neurological phenotypes may arise due to either increased or decreased p53 activity. Consistent with this notion, we speculate that the severity of the phenotype could depend, at least in part, on the intensity of the p53‐driven response induced by nonsense or missense SVBP variants.

Our findings prompt the question of whether senescence contributes to the pathophysiology of SVBP‐linked neurological disorders. Cellular senescence involves a loss of functionality and is implicated not only in normal aging but also in the etiology of several neurodegenerative diseases, including Alzheimer's disease (AD), Parkinson's disease (PD), frontotemporal dementia (FTD), amyotrophic lateral sclerosis (ALS), Friedreich ataxia (FRDA), multiple sclerosis (MS), and SARS‐linked complex spastic paraplegia (Carreno et al., 2021; Verdura et al., 2022). Therefore, we propose that cellular senescence could be the primary factors contributing to SVBP‐associated neurological disorders by promoting impaired progenitor proliferation, thereby impacting the regenerative capacities of the central nervous system (CNS) and rendering it susceptible to neurodegeneration.

Numerous factors observed in patients' cells, including centrosome alterations, cytokinesis defects, and resulting multinucleation and micronuclei formation, contribute to tumor growth (Fujiwara et al., 2005; Lens & Medema, 2019; LoMastro & Holland, 2019). In addition, the loss of p53 expression, accumulation of DNA damage, or a deficient immune system may enhance tumor invasiveness (Contreras et al., 2024; De Maeyer & Chambers, 2021; Marei et al., 2021). Notably, half of the patients carrying the pathogenic SVBP variant developed tumors in adulthood: patient P1 had adenocarcinoma with hepatic metastasis, patient P2 had a tubular adenoma, and patient P6 had breast cancer. VASH1, an endogenous angiogenesis inhibitor involved in various cancers, including colon and rectal cancers, has been shown to enhance tumorigenesis and metastasis in vivo when depleted (S. Liu et al., 2015). Our findings reveal that the SVBP knockout and consequent loss of VASH1 detyrosination activity promotes the proliferation of HeLa cells. Therefore, we propose that SVBP may play a crucial role in tumorigenesis and holds promise as both a diagnostic biomarker and a therapeutic target, warranting further research.

In summary, we describe a novel SVBP variant resulting in neurodevelopmental delay and complex HSP in several patients, and our molecular findings underscore centrosome defects and replicative senescence as major paradigms in the pathogenesis of neurological disorders.

## 4 EXPERIMENTAL PROCEDURES

### 4.1 Clinical studies

All patients were evaluated at the Neurology Department of Donostia University Hospital at different points in time, and the information was retrieved from clinical records. Patients and relatives gave written informed consent for the collection, storage, and publication of the clinical data, blood samples, and experimental analyses. The study was conducted in agreement with the Declaration of Helsinki and approved by the Clinical Research Ethics Committee of Bellvitge (PR076/14).

### 4.2 Whole‐exome sequencing (WES) and cosegregation studies

Genomic DNA was extracted from peripheral blood using standard methods. WES was performed on patient DNA samples from families A, B, and C using the SureSelect XT Human All Exon V5 50 Mb Kit (Agilent) for DNA capture and sequencing with the HiSeq 2000 Platform (Illumina) at CNAG (Centre Nacional d'Anàlisi Genòmica, Barcelona). We prioritized nonsynonymous coding variants that had a frequency lower than 0.01 in the ExAC, 1000 Genomes, and EVS databases. Candidate variants were validated and tested for cosegregation in all available family members by Sanger sequencing. The logarithm of odds (LOD) score was calculated with the MERLIN package using the variant genotype as entry data.

### 4.3 Cells and drugs

Primary human fibroblasts were collected from healthy individuals and patients (P2 and P4) according to IDIBELL guidelines for sampling, including informed consent from the persons involved or their representatives. Fibroblasts were prepared from skin biopsies. HeLa cells (ATCC® CCL‐2™) were obtained from ATCC. Human fibroblasts and HeLa cells were cultured in Dulbecco's modified Eagle's medium (DMEM; Gibco, Life Tech) supplemented with 10% fetal bovine serum (FBS) and 100 μg/mL penicillin–streptomycin at 37°C in a 5% CO2 atmosphere.

For PBMC extraction, blood samples from healthy individuals (29–64 years) and patients (P2 and P4) were collected in EDTA‐coated plastic tubes. PBMCs were separated by density gradient centrifugation using Histopaque (Sigma‐Aldrich, St. Louis, MO, USA). PBMCs were stored at −80°C until use.

The following drugs were used at the indicated concentrations: 2 μM for 3 h of paclitaxel (Sigma Aldrich) and 5 μM for 24 h of parthenolide (Sigma Aldrich). Control experiments were performed using the solvent DMSO (Sigma Aldrich).

### 4.4 Western blotting

Total cell extracts were prepared in RIPA buffer [50 mM NaCl, 1% Nonidet P40, 0.5% sodium deoxycholate, 0.1% SDS, 50 mM Tris, pH 8.0] supplemented with protease‐inhibitor mix (Roche) and Halt Phosphatase Inhibitor Cocktail (Thermo Scientific). Proteins were resolved by SDS‐PAGE using NuPAGE® Novex Bis‐Tris Gels (Invitrogen), transferred onto nitrocellulose membranes (Bio‐Rad) using the iBlot 2 Gel Transfer Device (Invitrogen) and analyzed with the required antibodies. A list of the antibodies used is provided in Supplemental Table S1. Proteins were detected with an enhanced chemiluminescence western blot detection system (GE Healthcare Bio‐Sciences AB) and visualized with the ChemidocTM Touch Imaging System (Bio‐Rad). Quantification of immunoblots was performed by densitometry using ImageLab Software (U.S. National Institutes of Health, USA).

### 4.5 Immunofluorescence

Cells were seeded on coverslips, fixed using 4% FA for 25 min, and washed with PBS. Next, cells were permeabilized and blocked in blocking buffer (1% BSA, 0.2% powdered milk, 2% NCS, 0.1 M glycine, 0.1% Triton‐X‐100) for 15 min at 25°C. The cells were incubated with primary antibodies at 4 °C ON, washed with PBS and incubated with secondary antibodies for 1 h at RT. A list of the antibodies used is provided in Supplemental Table S1. DNA was marked with DAPI (Sigma Aldrich). Confocal images were acquired using a Leica TCS SP8 STED 3X‐FALCON microscope (Leica Microsystems Heidelberg GmbH, Mannheim, Germany), and images were analyzed with ImageJ (NiH, USA).

### 4.6 RT‐ PCR

Total RNA was isolated from cells using the RNeasy Mini Kit (Qiagen), according to the manufacturer's instructions. Next, first‐strand cDNA was synthesized for each RNA sample using Superscript II reverse transcriptase (Invitrogen) and oligo‐dT. SYBR Green real‐time PCR was performed in the LightCycler® 480 Real‐Time PCR System (Roche Diagnostics GmbH, Mannheim, Baden‐Württemberg, Germany). Primers for human Lamin B1 (*lmnb1*; F‐5´‐AAGCAGCTGGAGTGGTTGTT‐3′, R‐5´‐TTGGATGCTCTTGGGGTTC‐3*′*), p*21* (F‐5′‐ctg gag act ctc agg gtc gaa‐3′, R‐5′‐cca gga ctg cag gct tcc t‐3′), and *p53* (F–5′‐aag aaa cca ctg gat gga gaa‐3′, R‐5′‐cag ctc tcg gaa cat ctc gaa‐3′) have been designed (Sigma Aldrich). Standardized primers for human *p16* (qHsaCEP0057827) and *Il6* (qHsaCED0044677) were used (Bio‐Rad). Expression of the genes of interest was normalized to that of the reference control human Rpl0 (Hs99999902). Each sample was run in duplicate, and the mean value was used to calculate the mRNA expression using the comparative (2 − ΔCt) method, according to the manufacturer's instructions.

### 4.7 Flow cytometry

For apoptosis assay, cells were washed and resuspended in binding buffer in the presence of APC Annexin and propidium iodide (APC Annexin V Apoptosis Detection Kit with PI, Biolegend). Cells were incubated for 15 min at room temperature and run in a BD FACSCanto.

For EdU incorporation, cells were pulse labeled with 10 mM EdU at 37°C for 45 min and fixed with 70% ethanol for at least 24 h at −20°C. Then, the cells were permeabilized with 0.5% Triton‐PBS 2 mM EDTA 1% FBS for 30 min and stained using the Click‐IT EdU Alexa Fluor 647 Imaging Kit (Invitrogen C10340) with some modifications. Finally, cells were resuspended in propidium iodide and run in a BD FACSCanto.

For H3S28P staining, cells were fixed with 70% ethanol and permeabilized with 0.5% Triton in PBS 2 mM EDTA 1% FBS for 30 min. To monitor mitosis, cells were stained with anti‐phospho Ser28 H3 antibody (BD Phosflow 558,217) for 3 h at room temperature. Finally, cells were resuspended in propidium iodide and run in a BD FACSCanto.

### 4.8 β‐Galactosidase activity

To determine senescent cells levels, the Senescence Cells Histochemical Staining Kit (Ref:CS0030, Sigma Aldrich) was used on fixed fibroblasts following product guidelines. Staining was examined with a Nikon Eclipse 80i microscope (Nikon, Japan).

### 4.9 CRISPR/Cas9 generation of *SVBP* ‐KO cell lines

gRNAs were selected (http://crispor.tefor.net/) and cloned into the psCas9(BB)‐2A‐GFP plasmid (Addgene PX458, 9.3 kb). Six different plasmids were transfected into HeLa cells using PEI (4 μL PEI/1 μg plasmid) (Table S1). Transfected cells expressing GFP were isolated by FACS, seeded in 96‐well plates (one cell per well) to obtain monoclonal lines, and incubated for 3 weeks. Clones were further expanded and characterized for protein depletion by PCR. The selected clone presents an inframe deletion of 24 bp, including the first coding methionine in exon 2 and a 75‐bp deletion in exon 3, starting at the last nucleotide of intron 2, thus affecting the canonical splice acceptor site of exon 3 and possibly inducing exon skipping. Western blot results confirmed the knockout of the SVBP protein (Figure S2c). As expected, SVBP‐KO HeLa cells exhibited lower levels of detyrosinated α‐tubulin after treatment with paclitaxel compared to control cells (Figure S2d).

### 4.10 Statistical analysis

Statistical significance was assessed using the Student's *t* test whenever two groups were compared. When analyzing multiple groups, we used one‐way ANOVA and Tukey's *posthoc* test to determine statistical significance. Data are presented as mean ± SD (*p < 0.05; **p < 0.01; ***p < 0.001).

## AUTHOR CONTRIBUTIONS

N.L. designed, performed, and analyzed the experiments, assembled figures and wrote the manuscript. E.V., J.O., and S.F. assisted in cell culture, immunofluorescences, transfections, screening of *CRISPR*/*Cas9* clones, and participated in manuscript writing. M.D.E. and A.V. designed and supervised cell cycle experiments and *SVBP*‐KO generation by CRISPR/CAS9 strategy and participated in manuscript writing. A.P. supervised the overall research, wrote the manuscript, secured the funding. MM and PLA assisted in confocal studies. M. R., G.F., P.I., A.R.P., and A.L.M performed the clinical studies and edited the manuscript.

## CONFLICT OF INTEREST STATEMENT

The authors declare that they have no conflicts of interest with the contents of this article.

## Supporting information


Appendix S1.

## Data Availability

The authors confirm that the data supporting the findings of this study are available within the article and/or its Supplementary material. Upon request, raw data can be made available.
