# Supplementary material for: Altered tubulin detyrosination due to SVBP malfunction induces cytokinesis failure and senescence, underlying a complex hereditary spastic paraplegia
Source: Aging Cell. 2024 Oct 16;24(1):e14355. doi: 10.1111/acel.14355 (PMC11709099; doi:10.1111/acel.14355)
Supplement: Supplementary file 1 — Appendix S1. [file ACEL-24-e14355-s001.docx]

SUPPLEMENTAL FIGURES


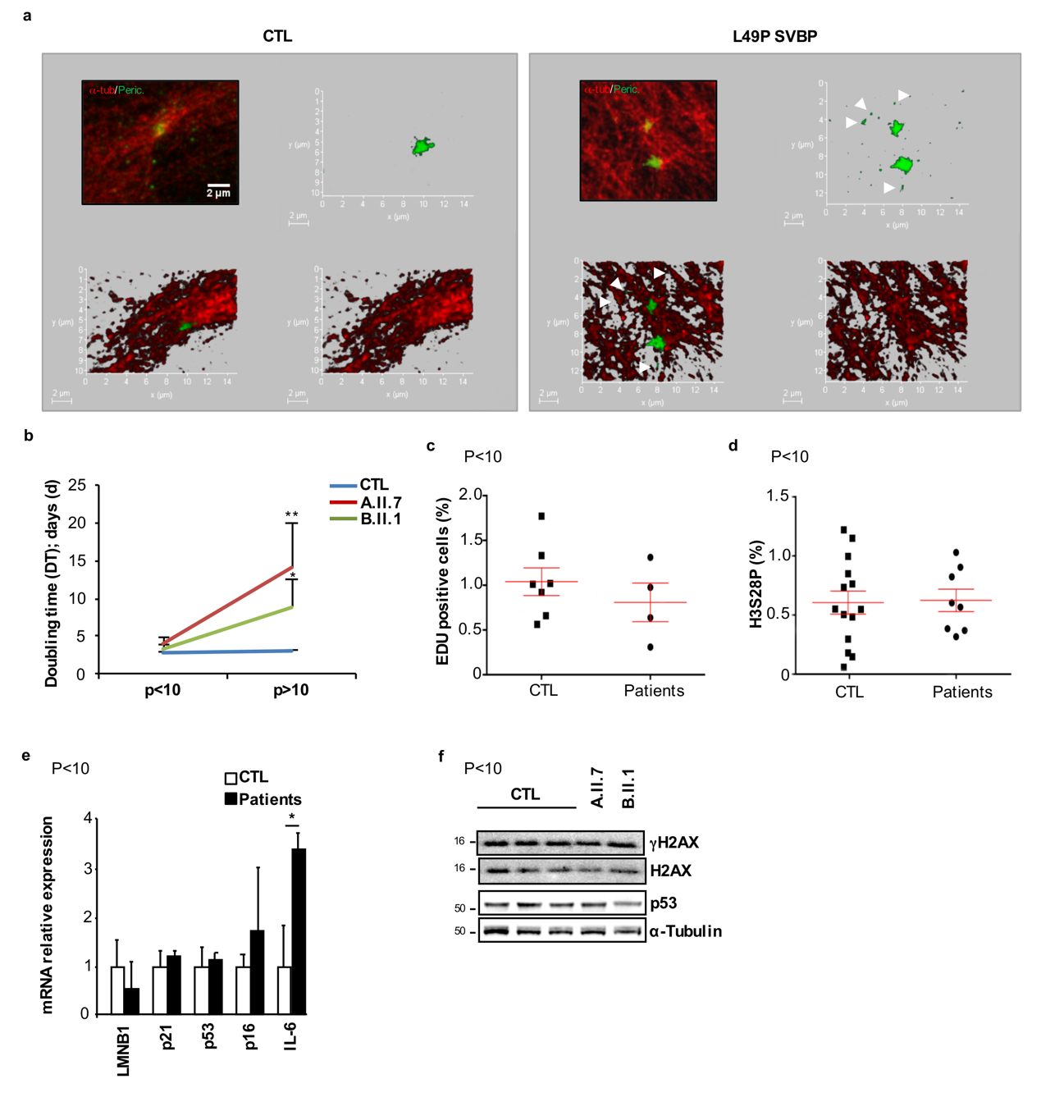


# **Figure S1:** (**a**) Representative 3D reconstruction of the microtubule (MT) cytoskeleton (α-tub, red) and centrosome (peric.; green) from control and patient fibroblasts at interphase. Scale bars, 2 µm. (**b**) Population doubling time (DT) of control (CTL) and patient fibroblasts (A.II.7 and B.II.1) according to the passage number. (**c, d**) Quantification of (**c**) EdU and (**d**) H3S28P positive cells from control (CTL) and patient fibroblasts (A.II.7 and B.II.1) at early passages (P <10)*.* (**e**) Quantitative RT-PCR analysis of *Lmnb1*, *p21*, *p53, p16* and IL-6 gene expression in control (CTL) and patient fibroblasts (A.II.7 and B.II.1) at early passages (P <10)*.* (**e**) Control (CTL) and patient fibroblasts (A.II.7 and b.II.1) at early passages (P < 10) were subjected to immunoblot analysis using anti-γH2AX, anti-H2AX and anti-p53 antibodies. Total amounts of α-tubulin were used as a loading control (n=2-3 per genotype).

**
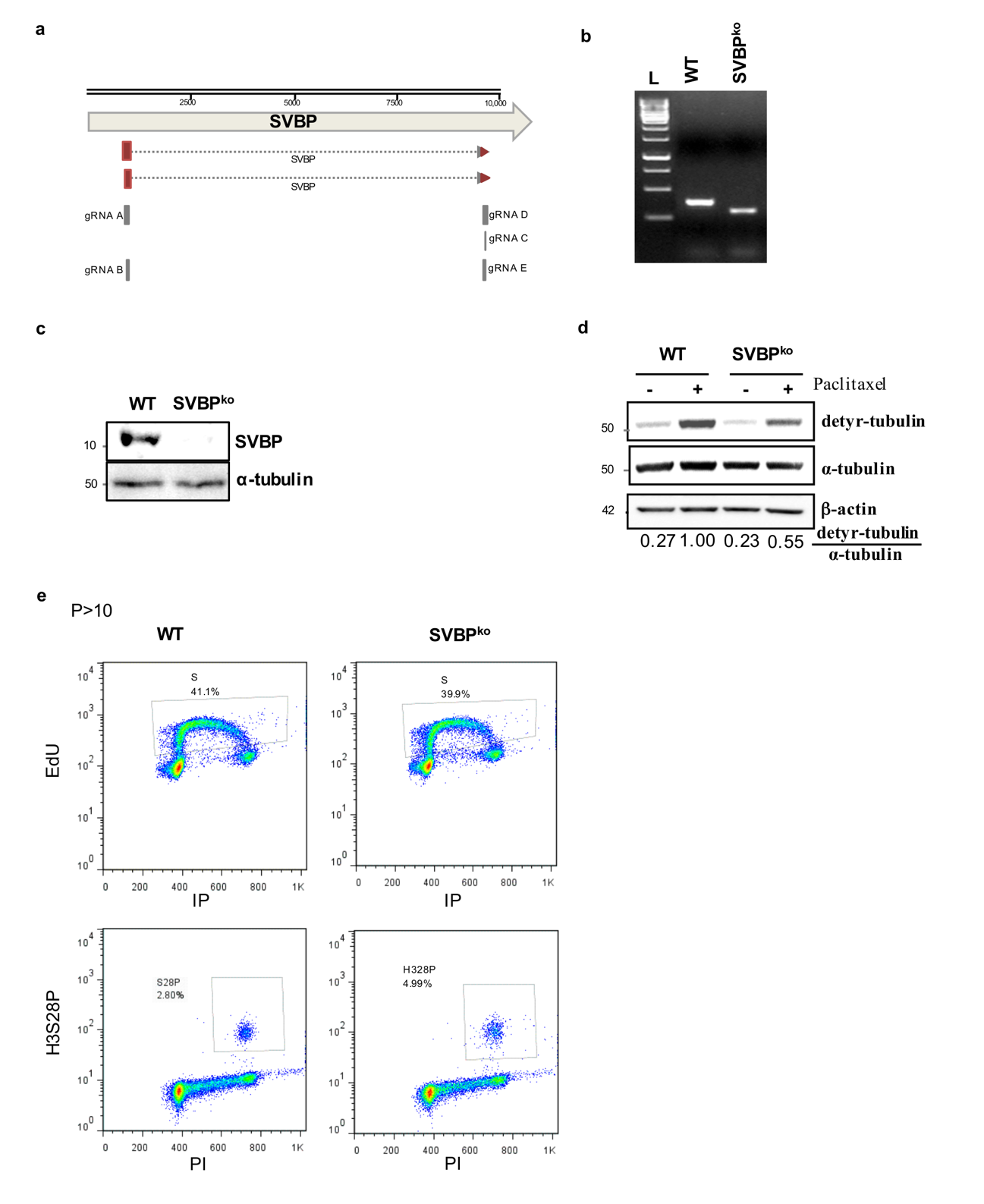
**

**Figure S2: (a-d) Construction and validation of the SVBP-KO HeLa cell line.** (**a**) Schematic representation of the strategy used to generate a SVBP-KO by CRISPR/CAS9-induced homologous recombination in HeLa cells. (**b**) PCR analysis of wild-type (WT) and SVBP-KO (SVBP^KO^) cell lines. Lane L, molecular ladder. (**c**) Wild-type (WT) and SVBP-KO (SVBP^ko^) HeLa cells were subjected to immunoblot analysis using the anti-SVBP antibody. Total amounts of α-tubulin (α-tub) were used as a loading control. (**d**) Wild-type (WT) and SVBP-KO (SVBP^ko^) HeLa cells were treated with vehicle or paclitaxel and subjected to immunoblot analysis using antibodies directed against detyrosinated (detyr-tubulin) and α-tubulin. Total amounts of β-actin were used as a loading control. The relative ratios of detyrosinated versus total ⍺-tubulin levels are indicated. (n=2 by genotype and condition). (**e**) Representative flow cytometry profiles of wild-type (WT) and SVBP-KO (SVBP^ko^) HeLa cells labelled with EdU and H3S28P at late cell passages (P >10).

**SUPPLEMENTAL TABLE**

|  | **Sequence (5’-3’)** | **Description** |
| --- | --- | --- |
| SVBP gRNA A F | tttacgtgcaggtggatcca | Exon 1 |
| SVBP gRNA B F | atctgtcagcagagttgaga | Exon 1 |
| SVBP gRNA C F | tgtaaacagatgcagcctcc | Exon 2 |
| SVBP gRNA D F | gttctgtcatgactctgttg | Exon 2 |
| SVBP gRNA E F | caacagagtcatgacagaac | Exon 2 |
| SVBP gRNA F F | gctgacagattctttaactt | Exon 1 |

T**able S1.** CRISPR-Cas9 guide-RNAs (gRNAs) used to generate knockout.
